# Narcolepsy: an interface among neurology, immunology, sleep, and genetics

**DOI:** 10.1055/s-0044-1779299

**Published:** 2024-04-02

**Authors:** Fernando Morgadinho Santos Coelho

**Affiliations:** 1 Universidade Federal de São Paulo, Departamento de Neurologia e Neurocirurgia, São Paulo SP, Brazil.

**Keywords:** Narcolepsy, Diagnosis, Comorbidity, Therapeutics, Narcolepsia, Diagnóstico, Comorbidade, Terapêutica

## Abstract

Narcolepsy is a primary disorder of the central nervous system resulting from genetic, environmental, and immunological interactions defined as excessive daytime sleepiness plus cataplexy, hallucinations, sleep paralysis, and sleep fragmentation. The pathophysiology is not entirely known, but the interaction among genetic predisposition, environmental exposition, and immune component with consequent hypocretin-1 deficiency is the model to explain narcolepsy type I. The mechanism of narcolepsy type II is less understood. There is a delay of over ten years for the diagnosis of narcolepsy around the world. Patients with narcolepsy have many comorbidities with a negative impact on quality of life. The treatment of narcolepsy must contain an educational approach for the family, coworkers, and patients. Scheduled naps and sleep hygiene are essential to minimize the dose of medications. Much progress has been seen in the pharmacological treatment of narcolepsy with new stimulants, different presentations of oxybate, and recent studies with orexin agonists. Narcolepsy is a rare disease that needs to be more understood and highlighted to avoid delayed diagnosis and severe disabilities in patients.

## Resumo

A narcolepsia é um distúrbio primário do sistema nervoso central resultante das interações genéticas, ambientais e imunológicas definidas como sonolência diurna excessiva mais cataplexia, alucinações, paralisia do sono e fragmentação do sono. A fisiopatologia não é completamente conhecida, mas a interação entre predisposição genética, exposição ambiental e componente imunológico com consequente deficiência de hipocretina-1 é o modelo para explicar a narcolepsia tipo I. O mecanismo da narcolepsia tipo II é menos compreendido. Há um atraso de mais de dez anos para o diagnóstico da narcolepsia em todo o mundo. Pacientes com narcolepsia apresentam muitas comorbidades com impacto negativo na qualidade de vida. O tratamento da narcolepsia deve conter uma abordagem educativa para a família, colegas de trabalho e pacientes. Cochilos programados e higiene do sono são importantes para minimizar a dose dos medicamentos. Muito progresso foi observado no tratamento farmacológico da narcolepsia com novos estimulantes, diferentes apresentações de oxibato e estudos recentes com agonistas de orexina. A narcolepsia é uma doença rara que precisa ser mais compreendida e destacada para evitar atrasos no diagnóstico e incapacidades graves nos pacientes.

## INTRODUCTION

Narcolepsy manifests with symptoms such as excessive daytime sleepiness (EDS), cataplexy, hypnagogic hallucinations, sleep paralysis, and sleep fragmentation. 1 The occurrences of cataplexy, hypnagogic hallucinations, and sleep paralysis are associated with REM sleep. 2 This condition is a primary central nervous system (CNS) disorder resulting from a complex interplay of genetic, environmental, and immunological factors. The prevalence is ∼0.02% in the general population. 3 Global investigations into narcolepsy prevalence have yielded similar results in regions such as Hong Kong, the United States, and Europe. 4 5 It does not display a gender predominance, with the highest incidence during adolescence and a secondary peak after the age of 40 in women, particularly around menopause. 6

Efforts have been invested in studies to elucidate the intricate pathophysiology of narcolepsy. A new finding is the reduction in hypocretin-1 levels observed in patients with narcolepsy and cataplexy, attributed to the disappearance of producer cells in the hypothalamus. Postmortem studies on narcoleptic patients affirm the loss of cells producing this neuropeptide in the lateral hypothalamus (∼50,000 to 100,000 cells). 7 8 A higher prevalence of the HLA-DQB1*0602 allele and a low hypocretin concentration in the central nervous system characterize individuals with narcolepsy and cataplexy. Although cataplexy is highly specific to narcolepsy, it can manifest in other clinical scenarios, including medication use and certain genetic diseases. 9

The diagnostic process can be challenging due to the influence of psychiatric disorders, clinical illnesses, shift work, sleep deprivation, and medication use, particularly in patients with type II narcolepsy. 10 This ambiguity in diagnosis may have adverse implications for individuals with narcolepsy in various aspects of their lives, including personal, professional, and legal domains. 11 This narrative review aims to comprehensively examine and discuss the current understanding of narcolepsy, specifically focusing on its clinical implications.

## HISTORICAL ASPECTS

Narcolepsy was initially recognized in 14 cases with uncontrollable sleep attacks by Gelineau 12 in 1889. Subsequent years saw the delineation of additional symptoms, with Lowenfield, Kinner, and Lhernitte describing cataplexy, hypnagogic hallucinations, and sleep paralysis in these patients, respectively. 13 In the second decade of the last century, while residing in Vienna amidst the Spanish flu outbreak, von Economo reported on EDS in patients with hypothalamus damage during infection with H1N1. 14

In 1930, Daniels coined the term “Gelineau tetrad” to describe the association of EDS, cataplexy, hypnagogic hallucinations, and sleep paralysis. 13 The understanding of narcolepsy's pathophysiology significantly advanced with Rechschaffen's electroencephalographic studies in 1967, which revealed sleep phases and exceptionally rapid eye movements (REM). 13

In 1973, narcolepsy was also described in Doberman dogs with an autosomal recessive or dominant Mendelian transmission pattern, depending on the canine species. 15 In the 1980s, a Japanese study demonstrated a higher prevalence of the HLA-DR2 allele in patients with narcolepsy. 16 In 1995, the association of Caucasian patients with narcolepsy and cataplexy with the presence of the HLA-DQB1*0602 allele in 95% of cases was described. 17

In 1998, the neuropeptide hypocretin or orexin, produced in the lateral hypothalamus with a regulatory function of sleep and appetite, was characterized. A few years later, a reduction in hypocretin-1 levels was evidenced in patients with narcolepsy. In 2005, the II International Classification of Sleep Disorders recognized sleep fragmentation as an element related to narcolepsy. 18 In 2010, an increase in cases of narcolepsy with cataplexy was linked to H1N1 vaccination in Asia and Europe. Studies have confirmed the association of narcolepsy with GSK vaccination with the adjuvant AS03. 19 20 The immunological theory was strengthened by identifying differences in patterns at the T lymphocyte receptor (TCR) locus and with specific tribbles homolog two antibodies described in patients diagnosed with narcolepsy in the first decade of this century. 21

EDS has been treated with typical stimulants such as methylphenidate since the 1960s. The evolution of treatment for narcolepsy will be discussed in a separate topic.

## GENETICS

The presence of the HLA-DQB1*0602 allele, a variant of the HLA-DQB1 gene, in the North American Caucasian population with narcolepsy and cataplexy is as high as 95%. However, the HLA-DQB1*0602 allele is not greater than 40% in patients without cataplexy. Due to this discrepancy, the HLA-DQB1*0602 allele is no longer used in clinical practice. 22 Nevertheless, the HLA-DQB1*0602 allele holds predictive value for individual variations in average sleep conditions, especially in sleep deprivation situations. 17 Fernandes et al. 17 demonstrated that the association between the HLA-DQB1*0602 allele and cataplexy could predict hypocretin-1 deficiency, offering potential utility in diagnosing diagnosis and treatment of narcolepsy.

In canine populations (Doberman and Labrador), autosomal recessive or dominant Mendelian transmission patterns have been confirmed depending on the breed. In 1999, the absence of hypocretin-2 receptors was confirmed as a crucial element in the pathophysiology of narcolepsy in these animals. Human narcolepsy does not have a Mendelian genetic transmission pattern. 15

## HYPOCRETIN (OREXIN)

Hypocretin, or orexin, is a neuropeptide produced in the lateral hypothalamus with two recognized receptors called 1 and 2 (using cyclic GMP with action on calcium and NMDA as a second messenger, respectively). Hypocretin affects sleep-wake modulation, pain, appetite control, and energy homeostasis. Hypocretin interfaces with the central nervous system (CNS) and the peripheral nervous system (dorsal root ganglion). 23 Human narcolepsy type I is characterized by low levels of hypocretin-1 following loss of hypocretinergic cells in the lateral hypothalamus. 24

Authors demonstrate that hypocretin exhibits seasonal and circadian variations, with increased levels of CSF during periods of attention or physical activity. Reduced or absence of hypocretin-1 levels drives volatility of the sleep-wake cycle with episodes of sleep attacks, hallucinations, sleep paralysis, cataplexy, and sleep fragmentation. 8

## PATHOPHYSIOLOGICAL HYPOTHESES OF NARCOLEPSY

The pathophysiological mechanism of narcolepsy remains incompletely understood, with various theories proposed. The degenerative theory advocates premature cell death of hypocretin-producing cells. Genetic theory associates narcolepsy with an increased familial predisposition of narcoleptic patients, as well as an earlier onset of signs and symptoms of narcolepsy in subsequent generations. It is worth noting that the prevalence of narcolepsy in children of a narcoleptic father or mother is less than 1%. 25 The environmental theory observes the interaction of the environment as physical, chemical, or biological agents with the loss of the affected cell population. The association between narcolepsy and infection by the H1N1 virus and after patients with rheumatic fever have been described. 19

However, the HLA-DQB1*0602 allele is more present in patients with narcolepsy and cataplexy, with a decrease in the population of hypocretinergic cells directly to an immunological mechanism. 17 26 Clinical improvement after using immunoglobulin and prednisone strengthens this hypothesis. 27 28 The association between narcolepsy and the TCR α locus, as well as the decrease in the concentration of soluble CD40 ligand (CD40L) in patients with narcolepsy, are strong indications of an autoimmune mechanism. 21 29

A recent publication reported the loss of specific immunological protection in the affected twin in a genome study of discordant twins. 30 The immunological theory would explain cell loss in the lateral hypothalamus in patients with narcolepsy due to self-harm due to an imbalance in the immune complex formed by TCR, HLA, and CD40L. 17 21 29 Furthermore, patients with narcolepsy with cataplexy have more specific tribbles homolog two antibodies, which, although present in all patients with narcolepsy, would be more prevalent in the initial phase of the disease. 31

The production of anti-hypocretin-2 receptor antibodies has also been described in patients who developed post-vaccination narcolepsy.

### Diagnosis and classification

**Figure 1:**
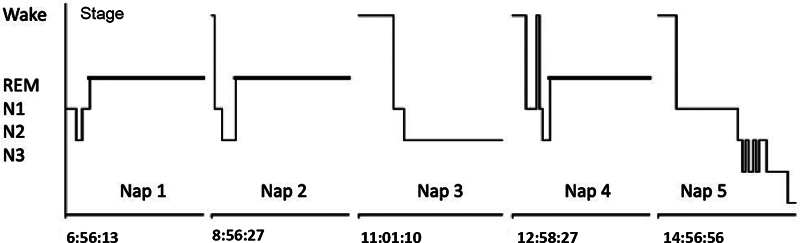
Multiple sleep latency test. Notes: 3 SOREMPS; Mean sleep latency – two minutes.

The diagnosis of narcolepsy is established by clinical and electrophysiological criteria and the level of hypocretin-1 in the CSF. Clinical criteria depend on the characterization of EDS associated with cataplexy, sleep paralysis, hypnagogic or hypnopompic hallucinations, and sleep fragmentation. 1 We used the Epworth Sleepiness Scale (ESS) to quantify EDS. 32 Scores above ten in Table 1 already characterize EDS. The electrophysiological diagnosis of the multiple sleep latency test (MSLT) is the study of five daytime naps for 15 minutes with intervals of 2 hours between each one. Figure 1 Typically, MSLT is preceded by all-night polysomnography (PSG), and the patient must be free from the use of stimulants or centrally-acting drugs such as antidepressants for at least four weeks or depending on the half-life of the medication being used. Prior PSG should ensure a minimum of 6 hours of sleep and the exclusion (alternatively ruling out) of other sleep disorders that trigger EDS. 1

**Table 1:** Epworth sleepiness scale

	0	1	2	3
1. Chance of dozing off while sitting and reading?	Never	Slight	Moderate	High
2. Chance of dozing off while watching TV?	Never	Slight	Moderate	High
3. Chance of dozing off while Sitting, inactive in a public place?	Never	Slight	Moderate	High
4. Chance of dozing off as a passenger in a car for an hour without a break?	Never	Slight	Moderate	High
5. Chance of dozing off while lying down to rest in the afternoon when circumstances permit?	Never	Slight	Moderate	High
6. Chance of dozing off while sitting and talking to someone?	Never	Slight	Moderate	High
7. Chance of dozing off while sitting quietly after lunch without alcohol?	Never	Slight	Moderate	High
8. Chance of dozing off while in a car, while stopped for a few minutes in traffic?	Never	Slight	Moderate	High
**Scores can be interpreted as follows:** 0–5 lower normal daytime sleepiness. • 6–10 normal daytime sleepiness. • 11–12 mild excessive daytime symptoms. • 13–15 moderate excessive daytime symptoms. • 16–24 severe excessive daytime symptoms.				

Source: Johns MW. A new method for measuring daytime sleepiness: the Epworth sleepiness scale. *Sleep* . 1991;14:540–5. PubMed ID: 179888.

The International Classification of Sleep Disorders (ICSD-3) classifies Narcolepsy into two types, I and II. 1 18

• Type I – Presence of cataplexy and/or CSF hypocretin-1 concentrations below 110 pg/mL or with a drop of ⅓ of the value of a previous dosage (children and adolescents starting the disease).

• Type II – Absence cataplexy and CSF hypocretin-1 concentration above 200 pg/mL.

The ICSD-3 defines the criteria for narcolepsy as MSLT with a mean latency of less than or equal to 8 minutes, in addition to two or more episodes of REM sleep during naps, possibly adding an early REM on the polysomnography that precedes the MSLT (less than 15 minutes). Recent revision of ICSD-3 recognizes the diagnosis of narcolepsy with characteristic clinical signs and symptoms of the disease with the presence of early REM in PSG. In cases of diagnostic uncertainty, repeating the MSLT may be a good option, just as another study demonstrates an exciting relationship between NREM and REM sleep, which characterizes an episode of REM sleep preceded and followed by NREM during naps in TMLS in patients with narcolepsy. 1 33 34

The presence of the HLA-DQB1*0602 allele is not part of the diagnostic criteria for narcolepsy and is performed for research purposes. Hypocretin-1 in the CSF should be measured whenever there is a clinical or electrophysiological doubt. Hypocretin-1 below 110 pg/mL or with a drop of ⅓ of the value of a previous dosage (children and adolescents starting the disease) is characteristic of type I narcolepsy. Patients with type II narcolepsy usually have levels of hypocretin-1, one greater than 200 pg/mL. 1 35

The literature should provide a more comprehensive discussion of the gray zone between 110 pg/mL and 200 pg/mL. Studies have reported cases of patients with hypocretin-1 levels within this range. Some exhibit the HLA-DQB1*0602 allele, along with cataplexy, sleep hallucinations, and sleep paralysis. Others, however, do not meet all the criteria for narcolepsy type I. While biomarkers are beneficial for identifying type I narcolepsy, identifying patients with narcolepsy type II remains challenging in many cases. 35

Unfortunately, in real life, there is a delay of over ten years for the diagnosis of narcolepsy around the world. 36 Many causes can explain this reality, such as the lack of knowledge on the disorder, incorrect diagnosis of depression, prejudice of familiar members and society about EDS, and few tools for clinicians. Recently, the Stanford Cataplexy Questionnaire and Narcolepsy Severity Scale were translated and validated for Portuguese in Brazil. 37 38

## COMORBIDITIES

Patients with narcolepsy often present with various comorbidities. Notable among these are obesity, a higher prevalence of psychiatric disorders, pain, olfactory impairment, fatigue, immunological diseases, and numerous side effects associated with medications, including an elevated risk of cardiovascular events. 39 40 41 42

The relationship between narcolepsy and psychiatric conditions has been a subject of extensive discussion. A recent study investigated the association of narcolepsy with schizophrenia (SCZ), major depressive disorder (MDD), and attention-deficit hyperactivity disorder (ADHD) using three distinct genome-wide approaches. The findings indicated a higher risk of SCZ associated with narcolepsy, a potential causal relationship between narcolepsy and MDD, while no significant relationship with ADHD was observed. 43

Despite daytime sleepiness being a critical criterion for diagnosing narcolepsy, individuals with this condition also exhibit a higher prevalence of fatigue. Patient-reported outcome measures have underscored a more frequent experience of fatigue in both types of narcolepsy, independent of sleepiness, depression, and obesity. 44

Researchers have reported a higher prevalence of endocrinological changes in teenagers with narcolepsy experiencing precocious puberty. 45 Managing these comorbidities is crucial to enhancing the well-being of these patients, directly impacting their overall quality of life. 11

Metabolic syndrome is particularly prevalent in children with narcolepsy. A retrospective study conducted in a French pediatric population revealed that 79.3% of narcolepsy patients exhibited high homeostasis model assessment for insulin resistance, 25.9% had an increased body mass index, 24.1% had low high-density lipoprotein cholesterol (HDL-C), and 12.1% had elevated triglyceride levels. Insulin resistance emerged as the central metabolic disturbance in both obese and nonobese children. Those with narcolepsy and metabolic syndrome also demonstrated more severe daytime sleepiness and a higher prevalence of night eating. 46

Patients with narcolepsy may encounter increased weight gain, olfactory changes, and a diminished quality of life. Studies have shown a more significant weight gain and abdominal obesity in patients with narcolepsy type 1, influenced by factors such as education level and olfactory function test scores. Data indicate that aging and hypocretin deficiency correlate with abdominal obesity, while years of study are primarily influenced by olfactory function. 40

Narcolepsy symptoms, coupled with concurrent medical conditions, adversely affect the daily activities of patients. Lower scores across all domains of quality-of-life questionnaires have been observed, particularly in narcolepsy patients type 1 and obese narcolepsy type II. Poor health status has been noted in Brazilians with narcolepsy type 2, suggesting that obesity negatively impacts physical domains. 47

Hypocretin-1 regulates nociception and pain in the central and peripheral nervous systems. Patients with narcolepsy exhibit a higher frequency of chronic pain. Depression is known to influence pain perception, and obesity may contribute to increased pain intensity in narcolepsy. 48 49

Recent evidence suggests a higher risk of bone fractures in patients with narcolepsy. 50 These individuals also show decreased cerebrospinal fluid (CSF) concentration of klotho and increased CSF levels of FGF-23, potentially predisposing them to a higher risk of osteoporosis and subsequent fractures. 51 52

## TREATMENT

The treatment of narcolepsy must be chosen individually and guided by standards recommended in modern guidelines in sleep medicine. 53 54 The regular follow-up of these patients in specialized centers guarantees the best result with the interaction of the multidisciplinary team. 53 54 55 A greater risk of accidents should always be highlighted, and potential risk situations should be avoided, even when using medication. Early identification and diagnosis provide better social and intellectual performance for these patients. The social and family integration of patients with narcolepsy must be guaranteed, with particular attention to depression and anxiety. Support and continuing education with information targeted at patients and their families are essential. 53 54

Patients should have good sleep hygiene with regular bedtimes and avoid the consumption of alcoholic beverages or other stimulating substances. In addition, scheduled naps of around 20 minutes and regular exercise improve complaints of daytime sleepiness. 53 54

Treatment aims to control EDS and cataplexy attacks. 55 56 Table 2 summarizes the main drugs available for these symptoms. Methylphenidate has been a therapeutic option for decades and is prescribed in the morning and after lunch in a daily dose ranging from 10 to 60 mg. 54 Lisdexamfetamine, available in the Brazilian market for treating ADHD like methylphenidate, has shown efficacy in treating narcolepsy in one study. However, further research is needed to establish the efficacy and safety of this drug. 57

**Table 2:** Pharmacological options to treat narcolepsy patients

Drug	Recommendation	Strengths of recommendation	Quality of evidence	Dosage	Side effects/notes
Modafinil	Excessive daytime sleepiness	Strong	Moderate	100–400mg	Headache, nausea, dry mouth, nervousness, dyspepsia, pain, vomiting, increased blood pressure, psychosis, and mania. # Risk of cardiovascular disorders. 56
Armodafinil	Excessive daytime sleepiness	Strong	Moderate	150–250mg	Headache, nausea, dry mouth, nervousness, dyspepsia, pain, vomiting, increased blood pressure, psychosis, and mania. # Risk of cardiovascular disorders. 56 57 58
Methylphenidate	Excessive daytime sleepiness	Weak	Low	5–60mg	Headache, nausea, dry mouth, nervousness, dyspepsia, pain, vomiting, increased blood pressure, psychosis, and mania. # Risk of cardiovascular disorders. 56
Amphetamines	Excessive daytime sleepiness	Weak	Low	Lisdexanfetamina 10–70mg	Headache, nausea, dry mouth, nervousness, dyspepsia, pain, vomiting, increased blood pressure, psychosis, and mania. # Risk of cardiovascular disorders. 56 62
Solriamfetol	Excessive daytime sleepiness	Strong	Moderate	37.5mg-150mg	Insomnia, headache, nausea, decreased appetite, anxiety. 56 Solriamfetol is not available in Brazil.
L-Carnitine	Excessive daytime sleepiness	Weak	Low	510mg	It is safe during pregnancy. 65 66 69
Selegiline	Excessive daytime sleepiness	Strong	Low	5–10mg	Dry mouth, headache, insomnia, sweating. 56
Caffeine	Excessive daytime sleepiness	Weak	Low	200mg	Complementary therapy. 70
Hypocretin receptor agonists	Excessive daytime sleepiness and cataplexy	No evidence	No evidence	–	A recent study was published with promissory results and liver toxicity. 61
Pitolisant	Excessive daytime sleepiness and cataplexy	Strong	Moderate	8.9–35.6mg	Headache, insomnia, and abdominal discomfort. 56 Pitolisant is not available in Brazil.
Sodium Oxybate	Excessive daytime sleepiness and Cataplexy	Strong	Moderate	1.5–9 g	Nocturnal confusion, enuresis, dizziness, nausea, weight loss, psychosis, depression, and worsening of OSA. 56 Oxybate is not available in Brazil.
Tricyclic antidepressants	Cataplexy	Strong	Low	Clomipramine 10–150mg	Weight gain, sexual dysfunction, constipation, and dry mouth. 56
Other antidepressants	Cataplexy	Strong	Low	Venlafaxine 37.5–225mg	Nausea, dizziness, decreased appetite, irritability, insomnia, weight gain, sexual dysfunction, hyperhidrosis, diarrhea, constipation. 56
Baclofen	Cataplexy	Weak	Low	10–40mg	Sleepiness, nausea, vomiting, diarrhea, headaches, dry mouth, blurred vision or difficulty focusing, excessive sweating, and rash. 64

We highlight the use of atypical stimulants known as modafinil and armodafinil, which are included in many guidelines around the world for narcolepsy treatment. 54 Modafinil improves wakefulness at 100 to 400 mg doses in adults but does not affect controlling cataplexy. Usually, we divide the total dosage by two times per day, in the morning and the last taken before 3 pm , to avoid insomnia. Recently, armodafinil has been available in Brazil. Armodafinil is prescribed once daily, in the morning, at 150 or 250mg. Armodafinil and modafinil both had a mean single-dose terminal elimination half-life (12 hours) and similar mean maximum plasma drug concentration (C(max)). However, after C(max), plasma concentrations decline in a monophasic manner with armodafinil and in a biphasic manner with modafinil due to the initial rapid elimination of its S-isomer. As a result, plasma drug concentrations are 33% and 40% higher, respectively, with armodafinil compared with modafinil on a milligram-to-milligram basis. 58 59

New therapeutic possibilities such as pitolisant (acting on histamine receptors) and solriamfetol (dual action - norepinephrine and dopamine) have been used to treat drowsiness complaints but are unavailable in Brazil. 60 61 A new class of drugs for narcolepsy is in an advanced testing phase, which is orexin two receptor agonists. Recent work demonstrates the benefit of this new class of medications in controlled studies, mainly in patients with narcolepsy type I but with liver toxicity. 54 62

Cataplexy can be treated with tricyclic and other classes of antidepressants such as citalopram, fluoxetine, and venlafaxine. The drug that has also been successfully used to control cataplexy is sodium oxybate or hydroxybutyric acid, which is not available in Brazil. 54 Recently, new presentations of sodium oxybate with a lower amount of sodium and a single nocturnal intake have been marketed in the United States of America. 63 Authors demonstrate the benefit of using baclofen in preventing cataplexy attacks. 64

An important point to be considered is related to the fact that the drugs used in the treatment of narcolepsy are Class C in pregnancy. 65 L-carnitine is an option to treat pregnancy patients. Authors have recently demonstrated partial improvement of symptoms after treatment with L-carnitine at the onset of symptoms, including pregnancy, in a systematic review. 66 67 Authors observed that the therapeutic efficacy of L-carnitine as a treatment for patients with narcolepsy in a population of 31 patients showed improvement in EDS. 68 Other authors have shown symptomatic improvement after using prednisone and immunoglobulin. 27 69 However, these treatments are experimental. They are under investigation, including a recent study that showed that caffeine, 200mg, can be a successful adjuvant treatment for treating EDS in narcolepsy patients. 70

Narcolepsy is a disease whose investigation integrates several areas of knowledge, such as neurology, immunology, sleep medicine, psychiatry, and genetics. 6 Patients with narcolepsy have personal, professional, and family impairments. 11 Many advances have been made in this exciting disease, an essential tool in disseminating information about the condition to medical colleagues and the general population. 11 New medications such as orexin agonists are promising to improve the quality of life of these patients. 62
